# The biological basis and clinical symptoms of CAR-T therapy-associated toxicites

**DOI:** 10.1038/s41419-018-0918-x

**Published:** 2018-09-04

**Authors:** Aleksei Titov, Alexey Petukhov, Alena Staliarova, Dmitriy Motorin, Emil Bulatov, Oleg Shuvalov, Surinder M. Soond, Mauro Piacentini, Gerry Melino, Andrey Zaritskey, Nickolai A. Barlev

**Affiliations:** 1 Almazov National Medical Research Centre, St. Petersburg, Russia 197341; 2 Institute of Cytology of the Russian Academy of Science, St. Petersburg, Russia 194064; 3 Institute of Fundamental Medicine and Biology, Kazan Federal University, Kazan, Russia 420008; 4 Belarussian Research Center for Pediatric Oncology, Hematology and Immunology, 223053 Borovliani, Republic of Belarus; 5 University of Rome Tor Vergata, 00173 Rome, Italy; 6 Moscow Institute of Physics and Technology, Dolgoprudny, Moscow Russia 141701

## Abstract

Currently, immunotherapy is attracting a lot of attention and may potentially become a leading approach in the treatment of cancer. One emerging therapeutic, the chimeric-antigen receptor T-cell adoptive immunotherapy (CAR-T) is showing remarkable efficacy in the treatment of several B-cell malignancies. The popularity of CAR-T has been founded on two CAR T-cell products recently approved by FDA (during 2017) in the treatment of relapsed/refractory B-cell acute lymphoblastic leukemia and B-cell lymphoma. However, their toxicities observed in clinical trials were extremely significant and in some cases even fatal with no approved algorithms for toxicity prediction being available to date. A deeper understanding of the biological basis of such complications is the key to prompt and comprehensive clinical management. Here we review the wide spectrum of effects associated with CAR T cell therapy with a major focus on the pathogenesis of cytokine release syndrome and neurotoxicity as the most common, potentially life-threatening effects of this treatment. We discuss the basis of clinical management and the existing models that predict the severity of toxicity, as well as the key factors that modulate this event. Finally, we will summarize the literature detailing universal allogenic CAR T-cells and their toxicity profile.

## Facts

1. The chimeric-antigen receptor T-cell adoptive immunotherapy (CAR-T) is a potent instrument for treating several hematological malignancies, not only those expressing the CD19 receptor.
2. There is a pressing need to make this therapy available to a wider spectrum of patients.
3. However, although the safety levels of CAR-T therapy are generally acceptable, several fatal outcomes due to severe cytotoxicity have been reported in clinical trials of CAR-T therapies.
4. Therefore, better understanding of the spectrum of toxicities, their etiology and pathogenesis as well as the knowledge of toxicity-promoting factors may help develop and validate the predictive scales and define better prophylactic strategies for high-risk patients.

## Open questions

It is known that some of the factors that worsen the toxicity of CAR-T therapy (higher CAR T-cell dose, intensive lymphodepletion) also positively affect its efficacy. How can one achieve the proper balance between these?

What kind of predictive model one should use for the toxicity risk assessment and which group of patients should be given the treatment for prophylaxis of such toxicity?

Would universal allogenic CAR T-cells be as safe and effective as the autologous CAR T-cells?

## Introduction

Adoptive immunotherapy is a rapidly evolving field in modern cancer biology and its treatment. This approach is based on the ex vivo modification and expansion of patient-derived antigen-presenting cells (APCs) or T-cells followed by their subsequent re-introduction back into the patient. One of the most promising modalities within this field is genetically modified T-cell expressing chimeric-antigen receptor (CAR-T), which is able to specifically recognize the target antigen (e.g., the CD19 receptor of B-cells) and eliminate the target cancer cells.

Although the safety profile of CAR-T therapy is generally acceptable and the treatment-related mortality is low, several fatal outcomes have been reported in CAR-T clinical trials. Better understanding of the spectrum of toxicities, their etiology and pathogenesis as well as the knowledge of toxicity-promoting factors may aid in the development and clinical validation of the predictive scales and define better prophylactic strategies for high-risk patients.

## Car design and therapeutic efficacy

### CAR structure

**Fig. 1:**
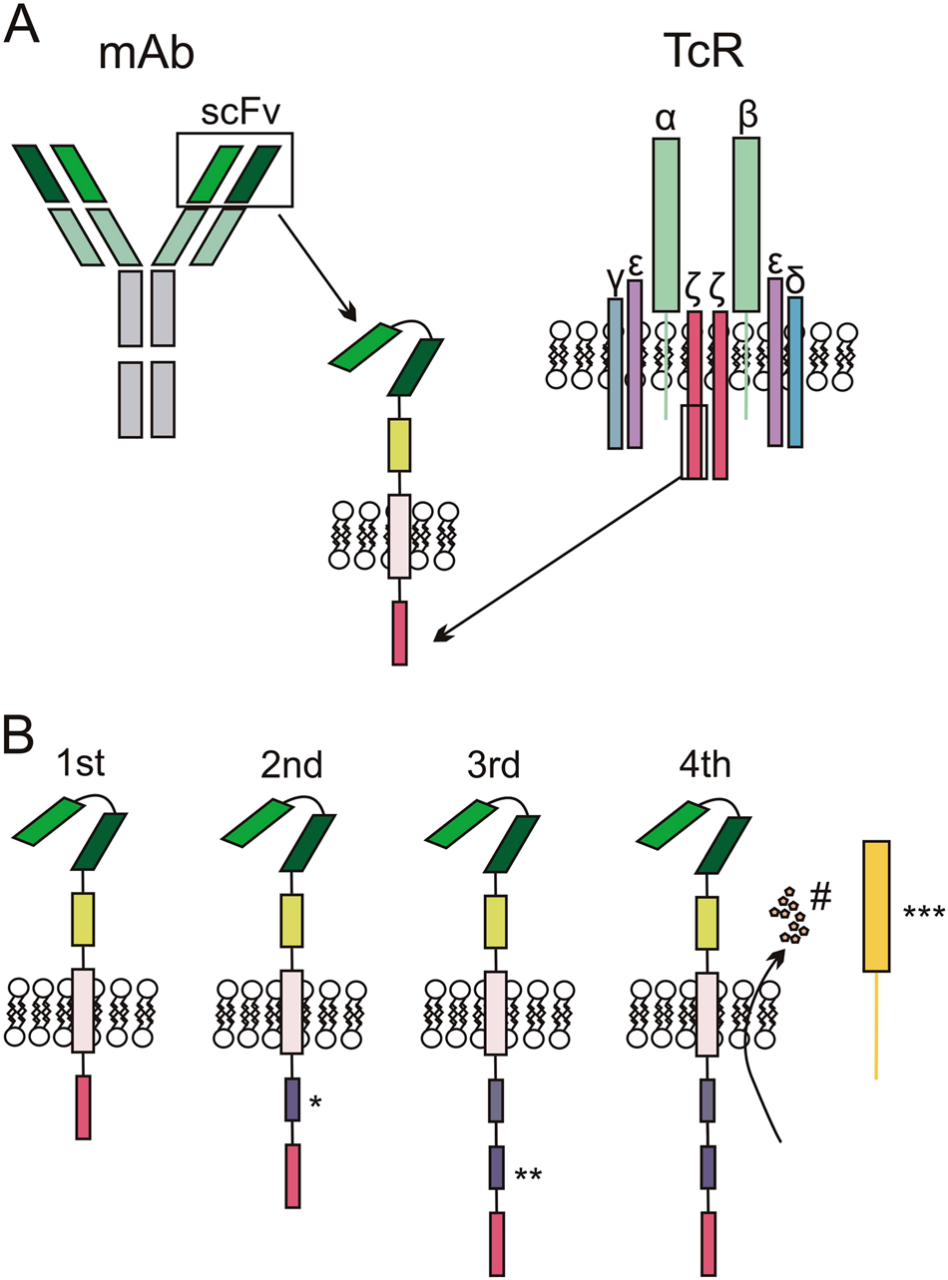
CAR and CAR-T design. **а** The basic design of CAR includes two functional parts: the extracellular domain (derived from the variable region of the monoclonal antibody (mAb) and reformatted into the single-chain variable Fragment (scFv) consisting of the linked variable regions of both heavy (V_H_) and light (V_L_) chains that recognize the target antigen, and the intracellular domain (derived from the ITAMs of the CD3 complex ζ-chain), providing the first activation signal. **b** The first-generation CAR consisted of scFv, transmembrane domain and CD3 ζ ITAMs as an intercellular activation signal. The second-generation CAR bears an additional co-stimulatory intracellular domain (*) such as CD28, CD137 signal domains. The third-generation CAR includes two or more different co-stimulatory domains (* and **) and the fourth generation CAR-T may be generally defined as the T-cell, bearing CAR of any design (2nd or 3rd generation) with expression of additional molecules, such as cytokines, antibodies (#) or separate co-stimulation molecules (***)

In general, CARs are transmembrane molecules composed of several functional elements. An extracellular single-chain variable fragment (scFv), derived from the antigen-recognizing sequence of an antibody, is fused to a hinge/spacer module and a transmembrane domain, which is further linked to the intracellular domain. The latter is critical for the transmission of the activation signal. CAR-T cells can been divided into four generations (as seen in Fig. 1). Whereas the term ‘generation’ was initially used to describe the CAR structure, it now more broadly (and collectively) refers to the CAR and CAR-T cells bearing it. The clinical trials that have yielded the most success include an FDA-approved second-generation CAR-T products from Novartis and Kite Pharma^1,2^. Third-generation CAR-Ts have also been explored and so far have failed to show additional benefits during clinical trials and consequently, further clinical studies are eagerly awaited^3^. More recently, a 4th generation of CAR-Ts has been developed and contains a genetic construct, encoding a separate co-stimulation molecule^4^ or suicide genes^5,6^, as well as cytokine genes^7^. The initial pre-clinical success^8^ of TRUCKs^7^, designed to additionally secrete IL12, could not be reproduced in clinical trials (NCT01236573, NCT01457131) and resulted in their termination due to unexpected toxicity and lack of effect.

### Therapeutic efficacy

CAR-T therapy has demonstrated the highest efficacy in the context of B-cell neoplasms. Despite the high rates of complete remission in acute lymphoblastic leukemia (ALL, up to 94% in large trials^1,6,9–11^), in one of the trials^9^ (phase I, CTL019, reviewed in Table 1), the probability of event-free survival was 67% at 6 months, and sustained remission was reported in 19 patients within a follow-up period of 1–24 months (with a median of 7 months). Most of the patients (15 of 19) received no further therapy. Of note, the results reported by Lee et al.^12^ and Davila et al.^13^, in the context of ALL, do not assess the curative potential of the therapy, as most of the patients proceeded to allogenic hematopoietic stem-cell transplantation (allo-HSCT) after achieving complete remission (CR). The latest articles improve our understanding of long-term outcomes reporting an overall survival rate of 52% at 18 months (ZUMA-1, median follow-up of 15.4 months; 42% of PTS showed a stable response)^2^ and 76 % at 12 months (ELIANA, median follow-up of 13.1 months)^1^. At the longest median follow-up of 29 months (range: 1–65), Park et al. (*n* = 53, ALL) reported the median event-free survival of 6.1 months (95% CI: 5.0–11.5) in which the median overall survival was 12.9 months (95% CI: 8.7–23.4). Importantly, a high disease burden, defined as ≥5% of bone marrow blasts or extramedullary disease, resulted in a significantly worse long-term prognosis^14^.

**Table 1:** CAR-T cell products, trials and articles used for the analysis of toxicities

Trial name	Patients (*n*)	Construct name, CAR-T design		Sponsor	Condition	Reference, comment	
ZUMA-1 NCT02348216	101	KTE019, Axicabtagene Ciloleucel^a^, CD28	FMC63 (scFv)	Kite Pharma	NHL	2,27^b^	
NCT01865617	29	JCART014, 4-1BB, defined CD4+:CD8+ cell ratio		Juno Therapeutics	ALL	11	28,61,95; Careful analysis of CRS, CRES and infections on the mixed population (133 patients)
	24			Juno Therapeutics	CLL	78	28,61,95; Careful analysis of CRS, CRES and infections on the mixed population (133 patients)
NCT01865617	32			Juno Therapeutics	NHL	79	28,61,95; Careful analysis of CRS, CRES and infections on the mixed population (133 patients)
NCT01626495 NCT01029366	30, phase I	CTL019, Tisagenlecleucel^a^, 4-1BB		Novartis	ALL	9	
ELIANA NCT02435849	75, phase II					1,26^b^	
NCT01044069	51, phase I	JCART015, CD28	SJ25C1 (scFv)	Juno Therapeutics	ALL	74,75; No grade 5 neurotoxicity observed	
ROCKET NCT02535364	38, phase II				ALL	96,97; Product development was terminated due to fatal neurotoxicity (5 deaths, cerebral edema)	

^a^FDA-approved drugs

^b^The article reporting the trial results and the FDA report necessary for drug approval

**Table 2:** MD Anderson Cancer Centre CRS grading system^31^

Symptom or sign of CRS	CRS grade 1^a^	CRS grade 2^a^	CRS grade 3^a^	CRS grade 4^a^
Vital signs				
Temperature ≥38 °C (fever)	Yes	Any	Any	Any
Systolic blood pressure <90 mm Hg (hypotension)	No	Responds to intravenous fluids or low-dose vasopressors	Needs high-dose or multiple vasopressors	Life-threatening
Needing oxygen for SaO2 >90% (hypoxia)	No	FiO2 < 40%	FiO2 ≥ 40%	Needs ventilator support
Organ toxicities^a^ Cardiac: tachycardia, arrhythmias, heart block, low ejection fraction Respiratory: tachypnea, pleural effusion, pulmonary edema Gastrointestinal: nausea, vomiting, diarrhea Hepatic: increased serum ALT, AST, or bilirubin levels Renal: acute kidney injury (increased serum creatinine levels), decreased urine output Dermatological: rash (less common) Coagulopathy: disseminated intravascular coagulation (less common)	Grade 1	Grade 2	Grade 3 or grade 4 transaminitis	Grade 4 except grade 4 transaminitis

^a^According to CTACAE v4.03

In relapsed or refractory acute myeloid leukemia (AML), the results were also quite promising^15,16^, although the absence of the ‘ideal’ AML-specific targets that are not expressed by normal hematopoietic progenitors may limit the scope of the CAR-T approach. Recent results of the BCMA-specific CAR-T therapy in heavily pretreated multiple myeloma (MM) patients showed particularly encouraging results. In this trial 95% CR/near CR with a median follow-up of 6 months^17^ were observed, although no data addressing the long-term survival is available.

In contrast to hematological malignancies, the efficacy of CAR-T cells is much lower for solid cancers. To date, the best results for CAR-T therapy in solid tumors have been reported for the treatment of HER2-positive sarcomas, with 4 of 17 PTS achieving disease stabilization^18^. Moreover, 3 of 11 neuroblastoma patients showed CR and two of them displayed prolonged CR^19^.

As there are several excellent reviews summarizing the recent information about clinical trials in the field of CAR-T^20–23^, we will omit the detailed description of clinical trial data in this review, to avoid duplication.

Despite the spectacular success of CAR-T therapy in treating B-cell malignancies, serious side effects have been associated with this therapy. As seen below, we will detail these adverse reactions and outline the clinical approaches currently being used to alleviate these complications.

## Toxicities of Car-T therapy

CAR-T therapy has a unique toxicity profile that is not easy to predict and evaluate as it may differ when distinct CAR-T designs are used. Consequently, unexpected toxicities were the reasons for why the early termination of several trials (e.g., NCT01236573, NCT01457131, and NCT02535364) had occurred. While this is a hindrance, many excellent studies are ongoing with a view to overcome this hurdle.

### Cytokine release syndrome (CRS)

CRS is the most common adverse event seen across the trials incorporating CAR-T cell therapy (74–100% in the anti-CD19 setting)^1,6,10,24–28^. This complication is thought to have arisen due to the expansion and activation of CAR-T cells which leads to a massive over-production of cytokines by a number of immune cell types that result in an elevated systemic inflammatory response.

#### Clinical manifestations of CRS

**Fig. 2:**
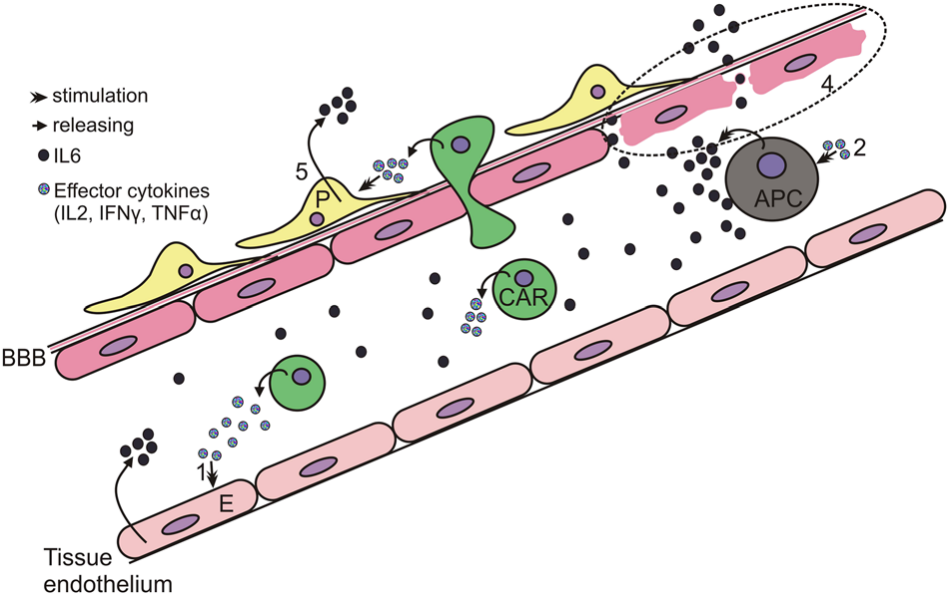
Pathogenesis of CRS and CRES. Activated CAR T-cells (CAR, green) release effector cytokines that in turn activate (1) Endothelial cells (E, pink) and (2) Antigen-presenting cells and macrophages (APC, gray). These cells produce IL6 driving CRS onset. (3) CAR-T cells are able to penetrate the blood–brain barrier (BBB). All the above-mentioned as well as other factors (see CRES pathogenesis) probably contribute to the BBB disruption and the passive passage of cytokines into the CNS (4) resulting in CRES. Pericytes (P, yellow) as well as endothelial cells exposed to effector cytokines produce IL6 (5) driving CRES further

As shown in Fig. 2, clinical symptoms of CRS can range from mild (fever, myalgias, fatigue, and mild hypotension) to serious symptoms, such as hypotension requiring vasopressors, respiratory failure, coagulopathy, and multi-organ system failure. CRS can manifest within the first week after CAR-T infusion and progress further within 1–2 weeks. Fever is usually the first and most obligatory sign of CRS^28^. Patients with more severe CRS experience the fever earlier which is prolonged and where the peak of temperature is higher^28,29^. Thus, the ‘time to fever’ and its peak have been adequately exploited in the CRS predictive scale^28^.

#### CRS grading

For prompt and efficient CRS management, accurate and robust clinical grading scales have recently been introduced, each with minor differences, where all of them categorize CRS from grade 1 toxicity that requires only symptomatic management to grade 4, which is potentially life-threatening, and grade 5, where lethal complications are evident. In clinical experiences, grade 4–5 CRS was noted as ‘severe CRS’ (sCRS).

Whereas, the grading of CRS originated from The National Cancer Institute Common Terminology Criteria for Adverse Events (CTCAE v4.03), then Lee et al.^30^ proposed the updated grading, and finally MD Anderson Cancer Centre CAR-T Therapy-Associated Toxicity Working Group (CARTOX) grading was developed^31^. The CTCAE grading was designed for CRS associated with antibody therapeutics, whereas Lee et al. developed the grading specifically for CAR-T therapy and defined low-dose requirement for vasopressors as grade 2 CRS. By contrast, the need for vasopressors was defined as grade 4 CRS in CTCAE. Furthermore, Lee et al.^30^ provided treatment guidelines based on their grading system and this grading system is most commonly used across the trials. Although the CARTOX scale seems to be nearly identical to that of Lee et al., it does have an advantage of it being easier to use (Table 2).

#### Pathogenesis of CRS

The first event driving CRS is the target-dependent activation of CAR-T cells, which release effector cytokines, such as IFNγ, TNFα, and IL2. These molecules in turn are able to activate macrophages that produce a broad spectrum of pro-inflammatory cytokines, resulting in hypercytokinemia and progression of CRS^32,33^. During CAR-associated CRS, the three most significantly elevated cytokines are IL6, IFN-γ, and IL10^34^. Their levels in CRS grade ≥4 increase ~2–3 logs in comparison to ‘no CRS’ cases^28^. Increased IL6 levels are associated with key clinical features of sCRS (which include hypoxia, hypotension, impaired coagulation and organ system failure)^35^. In clinical trials, anti-IL6-directed therapy was highly effective in CRS management^9,31,36,37^. Taken together, these data suggest that IL6 has a significant pathological role in CRS.

The IL 6 signaling pathway is exerted via the interaction of IL6 with its specific receptor (IL6R). The latter is present either in the membrane-bound form or in the soluble state (sIL6R). IL6R complexes the gp130 receptor, which serves a signal transducing component of the complex by activating JAK/STAT transcription pathway. Notably, IL6 signaling via the membrane-bound IL6R is restricted to hepatocytes, certain types of epithelial cells, and some leukocytes. This process is known as cis-signaling. On the contrary, during trans-signaling, sIL6R is excreted to serum where it binds circulating IL6 and, being recruited by the ubiquitously expressed gp130 component, may affect many tissues (reviewed in refs. ^38,39^).

Significant efforts have also been made to uncover the exact source(s) of IL6 during CAR-T cell activation^32^. Using co-culture assays (CAR T-cells and APCs) and data from the patients participating in clinical trials of CAR-T therapy, monocyte-lineage APCs were demonstrated to be exclusive IL6-producing cell type (among CAR T-cells, bystander T-cells). Based on the results of trans-well co-cultivation assays, it was importantly concluded that although occurring in response to CAR-T mediated recognition of leukemic cells IL6 production by APCs was independent of direct contact between CART19 and APCs^32^. Recent studies have explored endothelial activation in CRS and found that it may significantly modulate CRS severity. The markers for endothelial activation (VWF and Ang2) were elevated in patients developing sCRS, either before starting the CAR-T therapy or while sCRS developed and persisted^28^. A case study of a patient who succumbed to sCRS revealed endothelial cells as being one of the principle sources of IL6^40^, thus implicating a direct link between the activated endothelium to overall IL6 production. Notably, there is no evidence to date that T-cells or CAR-T cells may be a significant source of IL6^32^.

Importantly, IL6 was shown to influence only naive CD4+^41,42^ and CD8+ T-cells^43^. Upon activation, T-cells undergo a significant loss of IL6R density^41^, partly due to its increased shedding and hence formation of sIL6R^44^. Thus, although a problem of potential influence of IL6 on CAR T-cells cytotoxic function does exist, the likelihood of it is minimal^32^. This is because CD4+ and CD8+ CAR-T-cells function as activated T-lymphocytes that are no longer IL6- dependent. Indeed, IL6 was shown to have no impact on CAR-T-cell transcriptional profiling or cytotoxicity^32^. Moreover, it was demonstrated that CART19 treatment in an immune-deficient ALL mice model failed to mimic clinically observed CRS^32^, but was able to induce durable remissions in mice lacking APCs^45^, thus underlining the importance of immune cells (including APCs) interaction in pathogenesis of CRS. That blocking IL6 with the anti-IL6R antibody Tocilizumab did not significantly compromise therapeutic efficacy in clinics^31^, together with the above-mentioned data, suggest that IL6 is dispensable for CAR-T cell function. However, it should be mentioned that in the research by Singh et al.^32^ the level of sIL6R in the medium was not assessed thereby the absence of sIL6R-dependent trans-signaling in CAR-T should not be completely ruled out.

At present, it is not clear whether CD4+ CAR-T cells, similar to their normal CD4+ counterparts, are able to produce sIL6R and thus worsen the course of CRS. It should be noted however, that according to the work of Yang et al.^46^ the behavior of CD4+ CAR cells activated by CAR signaling was shown to be substantially different from CD4+ T-cells activated by TCR signaling. Based on this notion, it may be assumed that sIL6R secretion in CD4+ CAR-T cells also differs from the one in regular CD4+ T-cells. Clearly, this important question requires further experimental investigation.

**Fig. 3:**
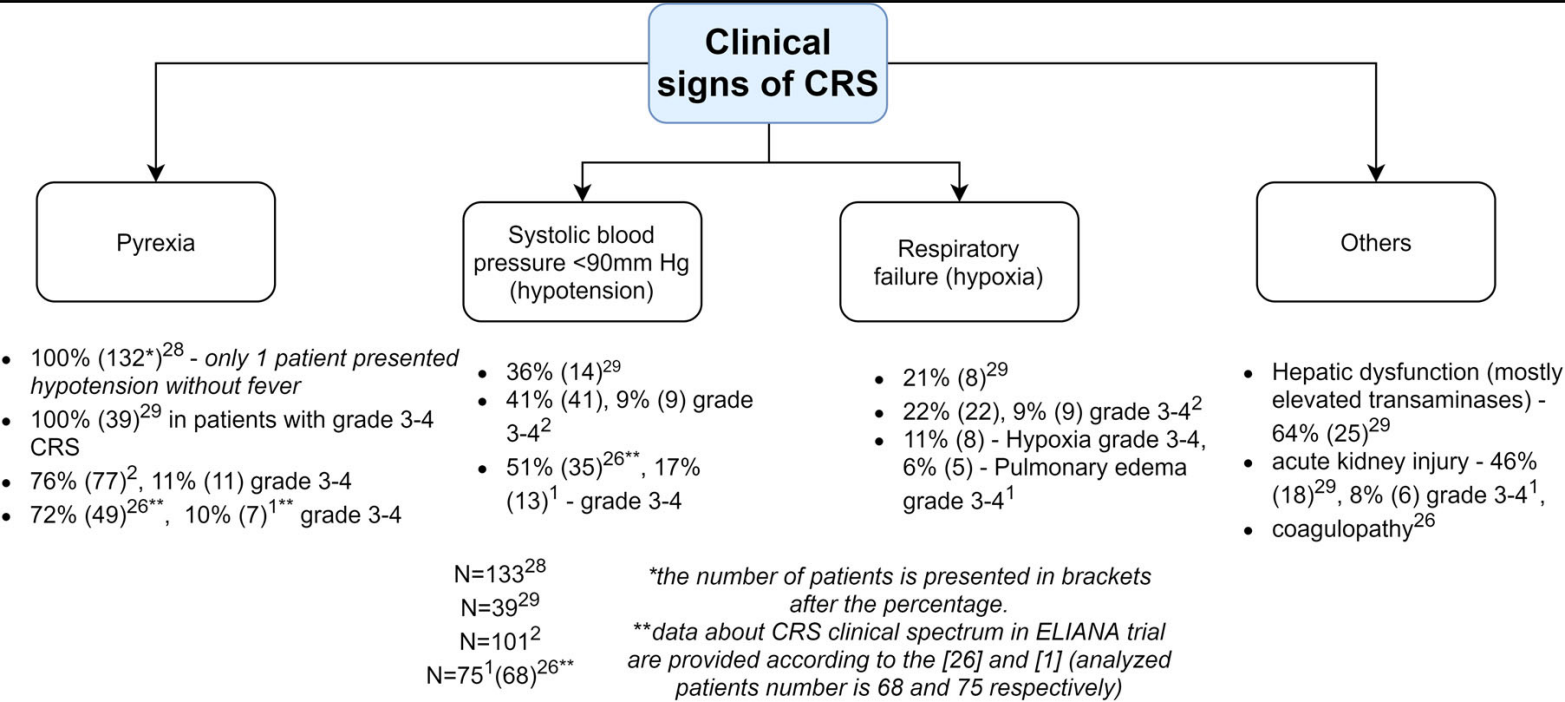
Clinical features of CRS according to the published data

As seen in Fig. 3, such events are depicted in the context of CRS.

#### Predicting CRS severity

Recently, Teachey et al.^47^ measured the levels of cytokines and biomarkers in 51 patients (of which 39 were pediatric), who were treated with CART19 (CTL019) for ALL. Therein, none of the standard clinical laboratory tests could predict CRS severity. Furthermore, many of the tests such as ferritin, CRP, LDH, AST, ALT, and BUN, showed abnormal levels only after CRS onset. Cytokine analysis has allowed the development of robust predictive models based on measuring 2–3 cytokines in children (such as IFNγ, IL13, and MIP1α) and adults (IFNγ, sgp130, eotaxin). Similarly, another prediction system has also been developed (from a study of 133 patients, JCAR014)^28^ based on the persistence of fever >38.9 °C during the first 36 h after therapeutic infusion. Here, sensitivity in sCRS prediction was 100% (with 84% specificity). Analysis of the information on a single cytokine (MCP-1) for patients with fever >38.9 °C allowed specificities of around 95% to be observed. In comparison to the model proposed by Teachey et al., this approach is more reliable, feasible and easier to follow as the measurement of MCP-1 values was needed from just 30 of the 133 patients, to safely classify the rest with a fever measurement. Although very encouraging, these approaches need further exploration through clinical trials. To ensure greater robustness one may suggest different CAR-T designs may ultimately also need design-specific CRS prediction scales.

#### HLH/MAS as a complication of CRS

Patients with sCRS may develop a macrophage activation syndrome (MAS), which also referred to as hemophagocytic lymphohistiocytosis (HLH). Identification of CRS-related MAS is complicated due to the commonality in pathogenesis of both conditions based on high serum cytokines such as IFNγ, which results in macrophage activation^48^. Outside the setting of CAR-T therapy, fever, cytopenia, hyperferritinemia as well as bone marrow hemophagocytosis are the key diagnostic features. To support the diagnosis, the exceptional diagnostic value of glycosylated ferritin for HLH/MAS has also been shown in several studies^49–51^. Following CART19 therapy, ferritin >10000 mg/dL (a typical diagnostic parameter for HLH in pediatric, but not in adult patients^52^) was invariably observed in all patients with grade 4–5 CRS and in 51 and 83% CRS grade 0–3 patients in pediatric and adult cohorts, respectively^47^. Thus, for patients treated with CAR-T, the only reliable indicators for HLH/MAS are hemophagocytosis, hypofibrinogenemia and probably hypertriglyceridemia, as the whole range of other above-mentioned diagnostic features is observed during CAR-T mediated CRS. Interestingly, hemophagocytosis in the absence of other HLH features has been noted to be non-specific for HLH diagnosis in adults^53,54^.

Recently, the frequency of HLH/MAS in CAR-T- treated patients has been reported to be as low as ~1%. The diagnostic criteria for this condition have been based upon unexplainable severity of liver, kidney and pulmonary organ toxicity and hemophagocytosis^31^. The treatment of HLH/MAS requires more active immune suppression and includes etoposide/cyclosporine-based regimens^31,55^. In this context, the diagnostic value of glycosylated ferritin has not been studied and may hold some potential.

### CAR-T-related neurotoxicity

Neurotoxicity, also known as CAR-T Related Encephalopathy Syndrome (CRES) is reportedly very common in CART19 trials^6,9,10,24,25^. The underlying cause is unknown and is likely unrelated to the recognition of cryptic CNS antigens by CART19 cells and off-target cytotoxicity. There is conflicting data about CD19 expression levels in brain tissue with more evidence for the absence of this antigen in the CNS^56–58^. Notably, neurotoxicity was reported in clinical trials of another CD19-targeting agent, Blinatumomab. CRES has not yet been clearly observed upon the targeting of other tumor antigens, except for CNS tumors^59^ and CD22 in ALL patients^60^. In the latter case, only mild subjective impairments were observed that might have been related to fever or comorbidity. Importantly, it must be noted that there were no cases of sCRS observed in this trial^60^ since only CRS grades 1 and 2 were reported, yet sCRS is known as high-risk factor for neurotoxicity (grade ≥3)^61^.

#### Clinical diagnosis and grading of neurotoxicity

**Fig. 4:**
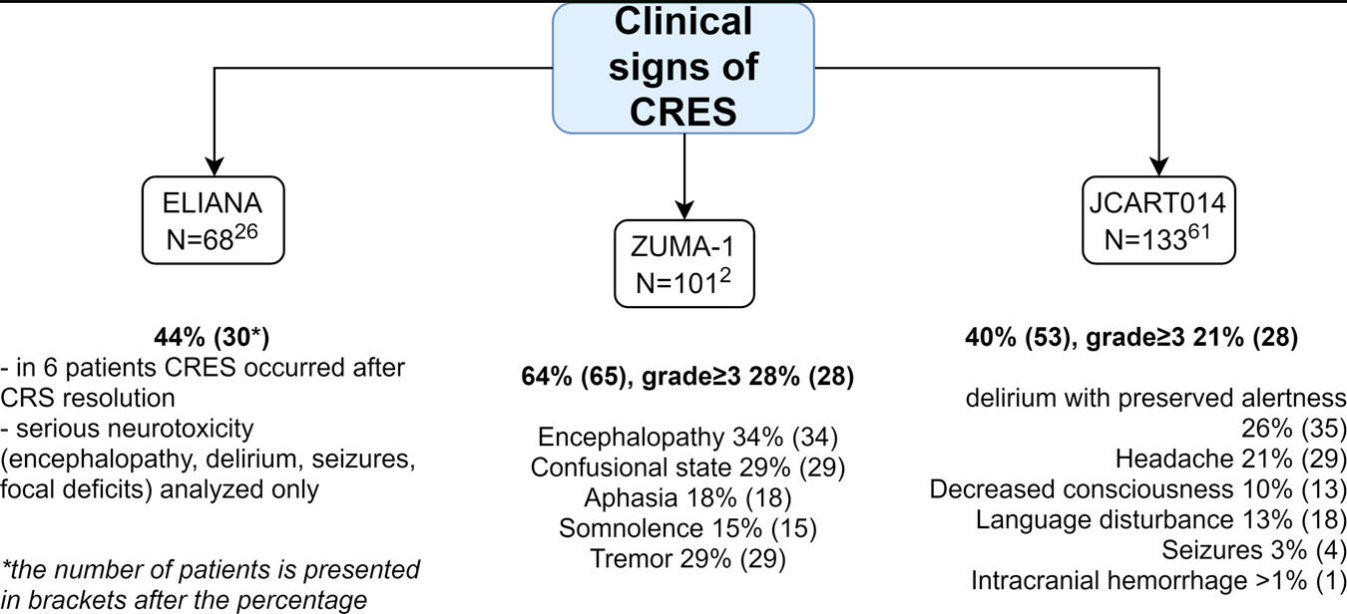
Clinical features of CRES according to the published data

Clinical symptoms of CRES (summarized in Fig. 4) commonly include headache, seizures, delirium, anxiety, tremor and impaired writing ability, aphasia, decreased consciousness and even coma with cerebral edema. The median time for onset of CRES is 4 days after infusion and the median duration is 5 days^61^. Grading of CRES is often performed according to CTCAE v. 4.03, but this system is imperfect, as it was not customized specifically for CAR-T neurotoxicity. Recently, an MD Anderson Cancer Centre CARTOX group has developed a new grading system (Table 3) based on a 10-point neurological assessment tool (CARTOX-10)^31^. It is very convenient and patients can be promptly assessed at high frequency. In brief, it evaluates patients’ ability to orient themselves in space, time and in their personality, as well it assesses their speech and writing abilities.

**Table 3:** MDACC CRES grading^31^

Symptom/sign	Grade 1	Grade 2	Grade 3	Grade 4
Neurological assessment score (by CARTOX-10)	7–9 (mild)	3–6 (moderate)	0–2 (severe)	Critical condition and/or obtunded and cannot perform assessment of tasks
Raised intracranial pressure	—	—	Stage 1–2 papilloedema^a^, or CSF opening pressure <20 mm Hg limiting self-care ADL	Stage 3–5 papilloedema^a^, or CSF opening pressure ≥20 mm Hg, or cerebral edema
Seizures or motor weakness	—	—	Partial seizure, or non-convulsive seizures on EEG with response to benzodiazepine	Generalized seizures, or convulsive or non-convulsive status epilepticus, or new motor weakness

^a^Papilloedema grading is performed according to the modified Frisén scale^98^

#### Pathogenesis and prediction of neurotoxicity

Several mechanisms for trafficking cytokines into the CNS have been previously described^62^. These include their passive leakage through the blood–brain–barrier (BBB), through active uptake mechanisms, via activation of endothelial cells and perivascular macrophages and on-site cytokine production downstream of the accumulation of immune cells in the CNS.

Following CART19 therapy, several of these mechanisms are engaged. First of all, CART19 cells penetrate the CNS as they have been detected in CSF^9,11,12,37,63,64^ either by PCR^63^ or by flow cytometry^11^. This is in line with the observations that CART19 therapy successfully eliminates CNS disease in patients with no subsequent CNS relapse^9,64,65^. Gust et al.^61^ found the concentrations of several cytokines (including IL6 and IFNγ) in CSF to be comparable with serum levels. This was interpreted as either a failure in the functionality of the BBB and/or due to the formation of local sources of cytokines in the CNS. Consistent with this idea, primary human BBB pericytes were demonstrated to secrete IL6 and VEGF upon exposure to IFNγ and TNFα. Furthermore, CNS toxicity was associated with increased BBB permeability mediated by the higher Ang2:Ang1 ratio in which Ang1 stabilizes endothelial cells of the BBB, while Ang2 has an opposing effect^61^. Interestingly, treatment of mice harboring experimental cerebral malaria (which is associated with a deregulated inflammatory response and high levels of TNFα, IL6, and IFNγ^66–71^), with fingolimod (a sphingosine-1-phosphate receptor modulator) was accompanied by higher Ang1 serum levels and decreased BBB permeability, which resulted in improved outcomes^72^. Similar observations were made for mice administered with recombinant Ang1^73^. Thus, pharmacologic modulation of the Ang2:Ang1 ratio could be explored further for acceptable CAR-T-associated CRES management^61^. In support of these findings, human autopsy results revealed the breakdown of the BBB in two patients who succumbed to CRES through cerebral edema after JCAR015 treatment^74^, highlighting the importance of this mechanism impact in severe CRES.

Neelapu et al.^31^ reported that the manifestation of CRES is biphasic with the onset of the first phase occurring simultaneously with CRS symptoms and usually within the first 5 days after CAR-T treatment. This ‘acute’ type of CRES had a tendency to be shorter and milder (grade 1–2) and may have benefitted from anti-IL6 therapy. The authors proposed that this may have been attributable to greater BBB permeability, which allowed the therapeutic antibodies to reach the CNS. An alternative explanation for the effectivity of anti-IL6 therapy was the lack of BBB damage during the ‘first phase’ of CRES, as the endothelium and pericytes were inactive and did not produce cytokines locally. In this instance, the elimination of IL6 after prompt therapy and resolution of CRS would prevent the progression of neurotoxicity by breaking the cytokine ‘loop’. Subsequently, the ‘second phase’ of CRES, which may happen even 3–4 weeks after cellular therapy (as seen in 10% of patients) may arise due to the formation of a local cytokine source within the CNS.

While CRS and CRES probably have certain commonalities in their pathogenesis, such as (1) IL6 production by APCs (and pericytes or endothelial cells) and (2) a deregulated endothelium (due to increased Ang2:Ang1 ratio and VWF), CRS and CRES are different pathological processes. In general, CRES has been rarely observed in the absence of any CRS, as evidenced in the study of CRES in the ZUMA-1 trial, in which 5 of 90 patients had CRES without CRS^27^ and in the Gust et al.^61^ patient cohort (where 5 of 53 patients had CRES with no CRS). These cases are usually mild and subjective (grade 1). However, there was also a case report of a patient with severe CRES with highly elevated CSF-cytokines in the absence of sCRS^63^. Taking into account the simultaneous prevalence of CRS and CRES grade ≥2^61^, CRS may appear to trigger the development of CRES, which may continue to develop independently (as highlighted in Fig. 3).

Gust et al.^61^ suggested that it would be advisable to prevent CRES development, as once established, it can be less likely to resolve after IL6-directed therapy. They proposed a predictive model for grade ≥4 CRES, based on a fever ≥38.9 °C and elevated serum IL6 and MCP1 levels in the first 36 h after CAR-T therapy. Here, the model had a sensitivity and specificity of 100 and 94%^61^. Alternatively, another group analyzing grade 3–4 CRES in 53 adults proposed a model based on the baseline platelet count of <60 (or a mean corpuscular hemoglobin concentration of >33.2%) and a morphological presence of ALL in bone marrow, which predicted grade 3–4 CRES with 95% sensitivity and 70% specificity^75^.

#### Management of CRS and CRES

Generally, the successful management of these significantly adverse events include addressing three key points. Firstly, careful patient evaluation before CAR-T infusion is necessary as there are some patient-related risk factors, such as thrombocytopenia for CRS^28,75^ and pre-existing neurological conditions^61^ for CRES (as well as a high tumor burden for both of them^28,61,75^. Secondly, close attention to fever as the first sign of CRS along with frequent monitoring for CRES clinical signs using the CARTOX-10 tool for both outpatients^30^ and inpatients are strongly recommended. Thirdly, a supporting treatment with anti-IL6 or steroid therapy according to refs.^30,31^ has to be prompt. In agreement with Neelapu et al.^31^, it is reasonable to start anti-IL6 therapy early in CRES when accompanied by CRS of any grade in order to prevent toxicity progression. In the prescription information for FDA-approved CAR-Ts that are listed in Table 1, the only IL6-directed therapy mentioned for the treatment of CAR-T-induced CRS is Tocilizumab (anti-IL6R). It should be noted that other agents, such as anti-IL6 (siltuximab)^76^, anti-TNFα mAbs (infliximab), soluble TNFα receptor (etanercept), and IL-1R-based inhibitors (anakinra)^30^ were also used. However, the current algorithm that employs Tocilizumab (with or without steroids treatment), allows the reversal of most cases of CRS within FDA-approved products, as no CRS-related deaths were reported in ELIANA trial and a low figure of 4% for the ZUMA-1 trial. Administration of Tocilizumab demonstrated prompt attenuation of CRS clinical symptoms (including fever and hypotension)^77^. However, this agent does not penetrate the CNS and may conversely increase CSF levels of IL6 and thus contributing to CRES development. Consequently, the anti-IL6 agent Siltuximab may be of preference (for neurotoxicity management), as it does not increase serum IL6 levels^61^. Thus current strategies for the treatment of CRS and CRES include anti-IL6 therapy which is generally more effective in CRS management, and steroids, which are often used to treat CRES.

The modern option for toxicity control may also include the use of suicide genes that are introduced into CAR-T cells for their quick elimination (if needed) and CAR T-cells of such design have already been tested in a clinical setting^6,11,78–80^. Additionally, a number of novel target-therapy approaches may also soon hold great potential in improving efficacy and toxicity management. For example, there is a pre-clinical study of CAR-T with specificity to diverse antigens secreting anti-PD1 antibody for efficacy improvement purposes^81^. As IL-6 is a cytokine associated with high toxicity (which apparently does not interfere with CAR-T cell function and therapy efficacy), developing CAR T-cells secreting the IL6 receptor with impaired function or an anti-IL6 antibody may therefore be key to safe and effective therapy, while simultaneously by-passing the need for anti-IL6 prophylaxis.

In summary, management of either CRS or CRES is challenging because both these complications are associated with high peak levels of CAR T-cells in the blood^28,61^. On the other hand, high concentration of CAR-T is required for efficient therapy and hence, decreasing CAR T-cell levels would compromise the treatment efficacy^28^.

Other side effects of CAR T-cell therapy are summarized in the Table 4.

**Table 4:** Adverse effects of CAR-T therapy

Adverse effect and definition	Frequency	Comments
Prolonged cytopenia (lasting > 30 days)	28%^27^ 32%^26^	Observed even in absence of lymphodepletion^37,99^ Probably due to CAR-T influence Resolved up to 6 months^26^
Hypogammaglobulinemia	15%^27^ 27–46%^95^	B-cell aplasia reported in 98% of PTS^95^ and is not necessary associated with hypogammaglobulinemia
Infections	22.6%^95a^ 14%^95b^	No difference in the rate of infections and their etiology compared to other anti-cancer therapies Bacterial^a^ and viral^b^ etiology predominated Risk factors—ALL, sCRS, CAR-T dose, number of prior therapy lines
Vector-associated complications: malignancy clonally related to modified cells (genotoxicity) or formation of Replication Competent Retroviruses (RCR)	—	RCPs reported in early studies^100^ Genotoxicity reported for other cell therapy^101–104^ even 15 years after treatment^105^ Not reported after CAR-T therapy probably due to (1) the use of vectors with less potent viral promoters (↓genotoxicity) and reduced recombination rate (↓RCR formation), (2) testing of cell product for RCRs, (3) lack of follow-up
Tumor lysis syndrome (TLS): metabolic disorder associated with massive release of tumor cell debris (hyperuricemia, hyperkalemia, hyperphosphatemia and hypocalcemia)^106^	4% (all grade 3)^1^ 14% (2/14 pts)^107^ 1 death^2^ 1 death^108^	Common in any type of anti-cancer therapy May contribute to other complications (e.g., acute renal failure due to CRS^109^) Risk factors—tumor burden, high proliferation rate, and highly responsive to treatment disease^110^ Multiple guidelines available^110–113^
Anaphylactic shock and anti-transgene immune response	0%^114^ 25%^115^	scFvs mostly derived from murine antibodies (immunogenic) Anaphylaxis case (IgE associated)^115^ observed in 1 of 4 PTS receiving multiple infusions of CAR-Ts. Not reported by another group (*n* = 47)^114^ Cases of anti-CAR antibodies^116^ or anti-CAR cellular response^11,117^ leading to decreased persistence and lack of clinical response
Graft-versus-host disease (GVHD) after donor-derived CAR-T infusion in PTS relapsed after allo-HSCT	—	No cases of GVHD^99,118^ 2 of 9 PTS experienced GVHD grade 3–4^108^

*PTS* patients

^a^0–28 and ^b^29–90 days after CART19 treatment

#### Main factors affecting toxicity

Analyzing the main factors that contribute to toxicity in CAR-T is complex, as previous trials have differed with respect to several features, such as CAR construct, disease type and lymphodepletion type. Consequently, we must be mindful of these differences particularly when comparing the outcomes from different trials. The factors affecting toxicity are summarized in Table 5.

**Table 5:** Factors associated with CAR-T therapy toxicity

Toxicity-contributing factors	Comments
Lymphodepletion regimen (chemotherapy given before CAR-T infusion)	Anti-transgene response was observed in the absence of lymphodepletion^116,117,119^ Combined lymphodepletion (Cy&Flu) resulted in better CAR T-cells expansion^11,120^, a higher serum CAR-T peak^61,75^ and higher toxicity (CRS and CRES)^28,61^ Combined lymphodepletion (Cy&Flu) was mentioned^96^ as the risk factor of fatal cerebral edema (ROCKET trial, see Table 1), however, after reverting back to monoCy lymphodepletion two more deaths were observed
Antigen type/epitope/scFv	Some of the tumor-specific CARs and TCRs are known to cross-react with normal tissue antigens (on-target off-tumor toxicities: B-cell aplasia in anti-CD19-therapies, cardiopulmonary toxicity in HER2^121^, and MAGE-A3-directed therapy^122^) Anaphylaxis and anti-CAR-T immune response are associated with murine epitopes in CAR^11^ Toxicity profiles may theoretically differ between the scFv domains due to their different affinities for specific epitopes of the target antigen JCAR015 (ROCKET trial, see Table 1) bore the recognition module derived from SJ25C1 in contrast to FMC63-based scFv used in other products by Novartis, Kite, and Juno Therapeutics. Toxicity impact unclear
CAR generation	Early trials with the first-generation CAR-Ts showed lack of both toxicity and efficacy^116,117,119^ Across second-generation CAR-Ts with different co-stimulatory domains the toxicity profile is very similar CD28-based CAR-Ts proliferate more actively and their peak expansion level is higher than that of the 4-1BB-containing CAR- Ts^96^ In turn, 4-1BB module ameliorates CAR-T exhaustion^123^ Little toxicity (low-grade CRS, no evidence of CRES) for the 4th generation 4SCAR19 bearing three co-stimulatory domains^6^. No comments on the toxicity profile are reported yet
T-cell subpopulation composition	Bulk CD8+ subset was an independent risk factor for CRS (JCAR014)^28^, as well as for severe CRES (JCAR015)^74^ JCAR014 and JCAR017 with defined CD4+:CD8+ composition^11,25^ are being developed by Juno Therapeutics JCAR017 demonstrated low rate of side effects (CRS and CRES)^124^, however, extended data are expected
Disease type	NHL appears to show less frequent CRS in comparison to ALL (30–57%^24,25^ vs 74–100%^6,10,26^ in the largest trials), however in ZUMA-1 (NHL) CRS incidence was 94% (39%—grade 1)^27^ For JCAR014, the type of disease impacted neither the severity of CRS nor CRES frequency^28,61^
CAR T-cell dose and expansion peak	Infusion of 5*10^8^ CAR T-cells resulted in unacceptable toxicity (all 6 patients developed CRS and 3 died). Splitting this dose over 3 days with flexible administration schedule resulted in 86% response rate and 66% CRS rate. 5*10^7^ cells dose resulted in efficacy decrease and comparable toxicity (*n* = 27, ALL)^125^ CAR T-cells dose was found to be a significant factor associated either with CRS and CRES^28,61^ For CRS, the interplay between CAR T-cell dose and Cy&Flu lymphodepletion was found, i.e., at any given CAR T-cell dose addition of fludarabine increased the probability of CRS^28^ onset Only weak association between severity of CRS and the peak of CAR T-cell expansion was shown (*n* = 51)^47^, but other data (*n* = 133)^28^ demonstrate the correlation of peak CAR T cell serum levels with both efficacy and toxicity of the therapy Logistic regression modeling performed to detect the therapeutic window^28^ balancing between toxicity and efficacy Serum IL15 levels are associated with higher CAR T-cells level^74,126^, efficacy of the therapy^126^ and ≥3 CRES risk^74,126^
Tumor burden	Borderline positive predictive value for sCRS (10 of 23 patients with >25% of marrow blasts developed sCRS)^47^, but strong negative predictive value (1 out of 15 patients with <5% bone marrow blasts experienced sCRS)^47^ In other studies, bone marrow blasts were included into predictive models^47,75^ for CRS and CRES The tumor burden-adapted treatment protocol was developed (JCAR014): the dose of 2*10^5^ CAR T-cells/kg for B-ALL with >20% marrow blasts; 2*10^6^ CAR T-cells/kg for B-ALL with ≤20% marrow blasts and for patients with NHL or CLL^11,78,79,95^

*PTS* patients, *Cy* cyclophosphamade, *Flu* fludarabine

## Future perspectives of Car-T approach

One important area of CAR-T research, which requires further development, stems from the limited clinical availability of CAR-T therapy. Considerations here include high costs of therapy and administration delays related to the time-periods required for manufacturing CAR-T. Due to the production failure that occurs in 5–10% of patients and other reasons mentioned above, the idea of designing a universal CAR-T (known as UCART) is an area of rapid development. For example, in 2012, Torikai et al. reported a plausible approach to designing UCART by modifying T-cells in such a way that the latter not only expressed the second-generation CAR, but also lacked the expression of the endogenous T-cell receptor. Here, the UCART was able to kill CD19+ cells and proliferate in vitro^82^. Another successful approach for generating UCARTs was reported in 2017 by Qasim et al.^83^ These UCARTs were provided by Celectis (France) and were designed to silence the expression of the TCRA and CD52 genes that allowed using Alemtuzumab (an anti-CD52 antibody) during the lymphodepletion regimen to prevent host immunity-dependent killing of UCARTs. The efficacy of this approach was observed in two infants with relapsed ALL. They were treated with these UCARTs without any signs of serious CRS and neurotoxicity. Encouragingly, both children reached CR and were negative for minimal residual disease and remained in CR after 18 and 12 months post-UCART administration.

Expanding the repertoire of antigen targets for CAR-T is another viable approach. In this respect, universal anti-CD123 CAR-T (UCART123) therapy initiated by Cellectis (2017) was not as successful as the CD19 one. The first two patients treated in two clinical trials for AML and blastic plasmacytoid dendritic cell neoplasm (BPDCN) experienced sCRS and capillary leak syndrome (CLS), which contributed to the death of one of the patients^84^. A clinical trial by Stemline Therapeutics, targeting the same CD123 with a monoclonal antibody demonstrated a similar toxicity profile as 3 of 32 patients experienced grade 5 CLS (resulting in their death). However, the therapy was hailed as generally effective^85^. At the same time, the phase I trial targeting CD123 with auto CAR-T cells in AML and BPDCN patients did not reveal any CLS cases^16^ and is therefore possible that the UCART123 toxicity profile (in CLS) could be attributable either to the allogenic origin of cells or to other yet unknown factors.

Alternatively, Celyad is developing a CAR-T bearing Natural Killer Receptor-2 that can potentially target several antigens both in solid and hematological malignancies. In a phase I clinical trial designed to treat AML and MM (NCT02203825), an acceptable safety profile was observed with no evidence of CRS, TLS or off-tumor/on-target toxicity during the first two (out of four) dose levels. At the same time, no objective responses were reported, although there was an improvement in overall survival after the therapy^86,87^. Currently, a phase I clinical trial which enrolled patients with different cancers is ongoing (NCT03018405) and indicates encouraging initial efficacy giving disease stabilisation for 2 out of 3 patients with colon cancer, whereas 1 AML patient experienced morphological CR^88,89^.

Furthermore, other ‘universal’ targets for CAR-T cells include VEGF-1 (and VEGFR2), which are not only expressed on vascular endothelial cells, but also on the tumor cells of different lineages^90^. While positive pre-clinical^91,92^, including suppression of metastasis^92^ were reported for CAR-T VEGF-1, the trials using CAR-T anti-VEGFR2 (NCT01218867) failed to show significant clinical activity.

## Conclusions

Undoubtedly, CAR-T therapy is one of the biggest recent breakthroughs in cancer therapy as it holds great potential and promise in the treatment of hematological malignancies and which may be an alternative to allo-HSCT for some patients^93^. In support of this, the long-term outcomes of patients who haven’t undergone allo-HSCT after CAR-Ts are eagerly awaited. At this moment, CAR-T therapy is still not fully functional against solid tumors and unfortunately, studies show little clinical benefit when extended into the clinical setting. As most of the pre-clinical models utilize immune-deficient animals, which fail to recapitulate the entire spectrum of interactions between immune cells, toxicities are therefore often observed in clinical trials for the therapies that were found to be safe in pre-clinical studies. To this end, great progress in understanding the molecular basis of toxicity has been instigated and which await further clinical validation. In support of this, experts in the field also agree on the necessity of developing customized prognostic scales for CAR-T-specific toxicity^94^, with several excellent scales having recently been reported^28,47,61,75^.

Looking forwards, indeed more work needs to be done to unravel the full potential of CAR-T-based anti-cancer therapy, but the clinical results of this therapy, achieved to date, offer great optimism and therefore further investigations are certainly justified and the outcomes of which are eagerly awaited.
